# Natural killer cell activation contributes to hepatitis B viral control in a mouse model

**DOI:** 10.1038/s41598-017-00387-2

**Published:** 2017-03-22

**Authors:** Shiwen Tong, Guangze Liu, Minghong Li, Xiumei Li, Qian Liu, Hong Peng, Shiying Li, Hong Ren, Wenwei Yin

**Affiliations:** 1 Key Laboratory of Molecular Biology for Infectious Diseases (Ministry of Education), Institute for Viral Hepatitis, Department of Infectious Diseases, The Second Affiliated Hospital, Chongqing Medical University, Chongqing, China; 2 Department of Clinical Nutrition, The Second Affiliated Hospital of Chongqing Medical University, Chongqing, China; 3 Center of Infectious Diseases, 458th Hospital of PLA, No. 801 Dongfengdong Road, Guangzhou, China

## Abstract

The roles of CD4 + T cells and CD8 + T cells in hepatitis B virus (HBV) infection have been well documented. However, the role of innate immunity in HBV infection remains obscure. Here we examined the effect of activation of innate immunity by polyinosinic: polycytidylic acid (PolyI:C) on HBV infection. A chronic HBV replication mouse model was established by hydrodynamical injection of pAAV/HBV1.2 plasmid into C57BL/6 mice. We found that HBV did not seem to induce an active NK-cell response in the mouse model. Early PolyI:C treatment markedly decreased serum HBV levels and led to HBV clearance. Following PolyI:C injection, NK cells were activated and accumulated in the liver. Depletion of NK cells markedly attenuated the anti-HBV activity of PolyI:C. Moreover, we found that IFN-γ production from NK cells was essential for the antiviral effect of PolyI:C in the model. Importantly, activation of NK cells by PolyI:C could also lead to HBV suppression in HBV-tolerant mice and HBV-transgenic mice. These results suggest that activated NK cells might suppress HBV and contribute to HBV clearance during natural HBV infection. In addition, therapeutic activation of NK cells may represent a new strategy for the treatment of chronic HBV infection.

## Introduction

The hepatitis B virus (HBV) is a noncytopathic, hepatotropic DNA virus that causes acute and chronic hepatitis often leading to liver cirrhosis as well as hepatocellular carcinoma^1^. The chance of clearing HBV infection is dependent on the age of HBV exposure. Ninety-five percent of adult-acquired infections lead to spontaneous clearance, whereas up to 90% of exposed neonates fail to resolve HBV and develop chronic infection^2^. The outcome of HBV infection in humans (viral clearance or viral persistence) is determined by complicated, as yet not fully understood, interactions between HBV and the immune system^3^. Both innate and adaptive components of the immune system mediate protective immunity against a viral infection, with innate responses being important for limiting viral replication and spreading very early after infection, as well as for a timely orchestration of virus-specific adaptive responses^4^. The liver is considered as an innate immune organ as it is highly enriched with innate immune cells that play a critical role in orchestrating the body’s host defense^5^. Surprisingly, HBV appears to act as a “stealth virus” and does not induce a measurable innate immune response in the infected liver^6, 7^, it seems to employ active strategies to evade innate immune responses to achieve persistent infection^8–10^. We speculate that boosting of the innate immune response during the early phase of HBV infection may be useful in reducing viral spread and preventing HBV persistence.

Natural killer (NK) cells have been viewed as the most important effectors of the initial antiviral innate immune system^11^. Compared with their relatively low frequency in the peripheral lymphatic system, NK cells are highly enriched in the liver^12, 13^, the site of HBV replication, implying that HBV has to evade NK cell-mediated immune responses to establish a persistent viral infection^14^. Based on the abundance of NK cells in the liver and their ability to produce antiviral cytokines, it is possible that activated NK cells might inhibit viral replication during HBV infection. To test this hypothesis, in the present study, we attempted to elucidate the role of NK cells in HBV infection by monitoring the ability of PolyI:C, a potent stimulator for NK cells^15^, to control HBV infection.

In the past few years, most *in vivo* studies on the mechanisms of HBV tolerance have been approached by using HBV transgenic mice which are inherently tolerant to HBV virus and have limitations in addressing what happens at the onset of HBV infection that influences the final outcomes of HBV infection^16, 17^. Recently, a nontransgenic mouse model of HBV tolerance was established by hydrodynamic injection (HI) of the plasmid pAAV/HBV1.2 into immunocompetent mice^18^. The characteristics of this mouse model are analogous to those of human chronic HBV infections. In the current study, we used this model to examine the effects of PolyI:C on HBV infection. We found that PolyI:C treatment could control HBV infection in a NK cell and IFN-γ-dependent manner.

## Results

### Establishment of a HBV-tolerant mouse model

**Figure 1:**
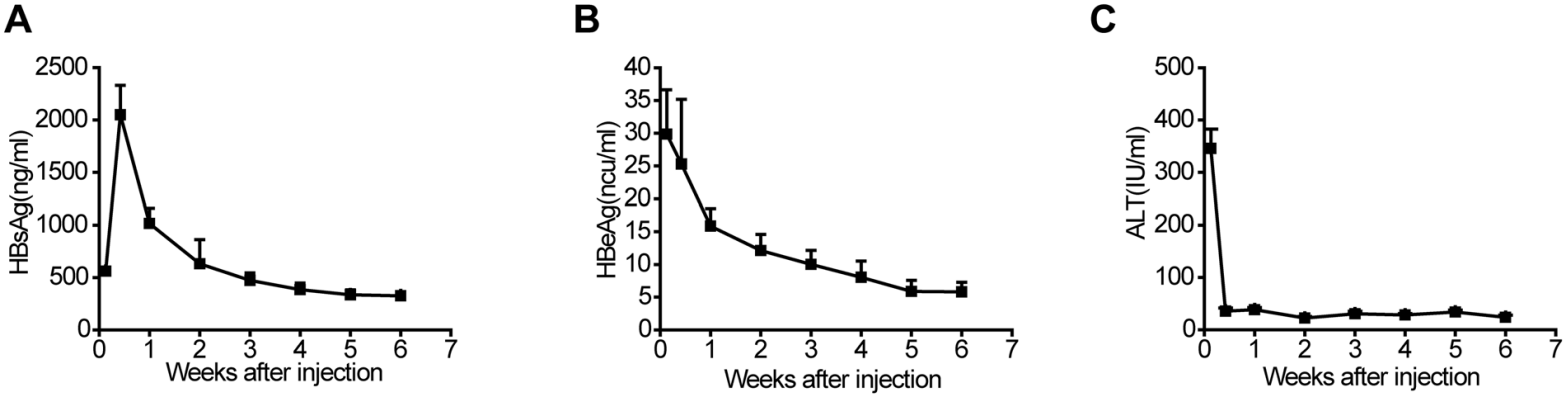
Hydrodynamic injection of pAAV/HBV1.2 plasmid leads to the persistence of HBV. (**A**,**B**,**C**) C57BL/6 mice received 6 μg of pAAV/HBV1.2 plasmid through hydrodynamic injection. Serum HBsAg (**A**), HBeAg (**B**) and ALT (**C**) levels were detected at different time points after injection. Data are shown as mean ± SEM, *n* = 10.

To address whether activation of innate immune system could influence the final outcomes of HBV infection, we utilized a nontransgenic HBV-carrier mouse model developed by hydrodynamic injection of the pAAV/HBV1.2 plasmid into C57BL/6 mice^19^. Serum HBsAg (Fig. 1A) and HBeAg (Fig. 1B) can be detected 1 day post pAAV/HBV1.2 injection and remained high 6 weeks post injection. There were no detectable serum anti-HBs in these animals (data not shown). Serum alanine aminotransaminase activity (ALT) increased on day 1 due to the hydrodynamic procedure and then returned to baseline levels thereafter (Fig. 1C), suggesting no hepatitis flare following pAAV/HBV1.2 injection. Thus, hydrodynamic injection of pAAV/HBV1.2 leads to a persistent HBV gene expression in mice, which mimics the immune tolerant phase of chronic HBV infection in humans.

### NK cells remain inactivated in the HBV mouse model

**Figure 2:**
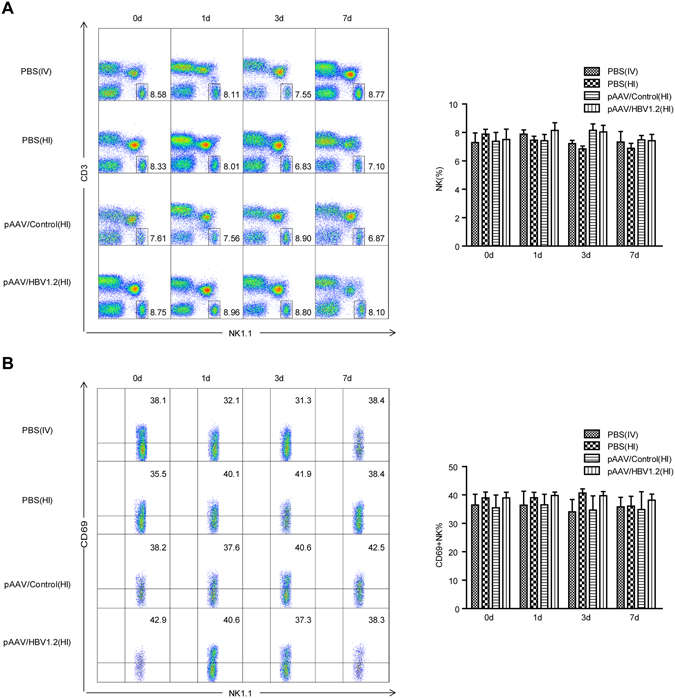
HBV does not seem to induce an active NK-cell response in the mouse model. C57BL/6 mice received 6 μg of pAAV/HBV1.2 plasmid through hydrodynamic injection. Mice received HI of pAAV/control plasmid or HI of PBS or intravenous injection (IV) of PBS were used as controls. Lymphocytes were isolated from the liver at the indicated time points after injection. (**A**) The percentages of NK cells (CD3-NK1.1+) were analyzed using anti-NK1.1 and anti-CD3 antibodies. (**B**) The surface expression of CD69 on gated NK cells (CD3-NK1.1+) was also analyzed. Data are shown as mean ± SEM, *n* = 4.

We first examined the number and activation status of NK cells in pAAV/HBV1.2-injected mice compared to control mice. Considering that HI of DNA plasmid may induce innate immune responses, mice received HI of pAAV/control plasmid or HI of PBS or intravenous injection (IV) of PBS were used as controls. We found that the percentage of hepatic NK cells did not significantly change after pAAV/HBV1.2 injection (Fig. 2A). Also, the expression of the early activation marker CD69 on hepatic NK cells was not increased in HBV-injected mice compared to all the control groups (Fig. 2B). These data indicated that HBV did not elicit a strong NK-cell response in the HBV mouse model, which may be favourable for HBV replication and persistence.

### Early PolyI:C treatment leads to HBV clearance

**Figure 3:**
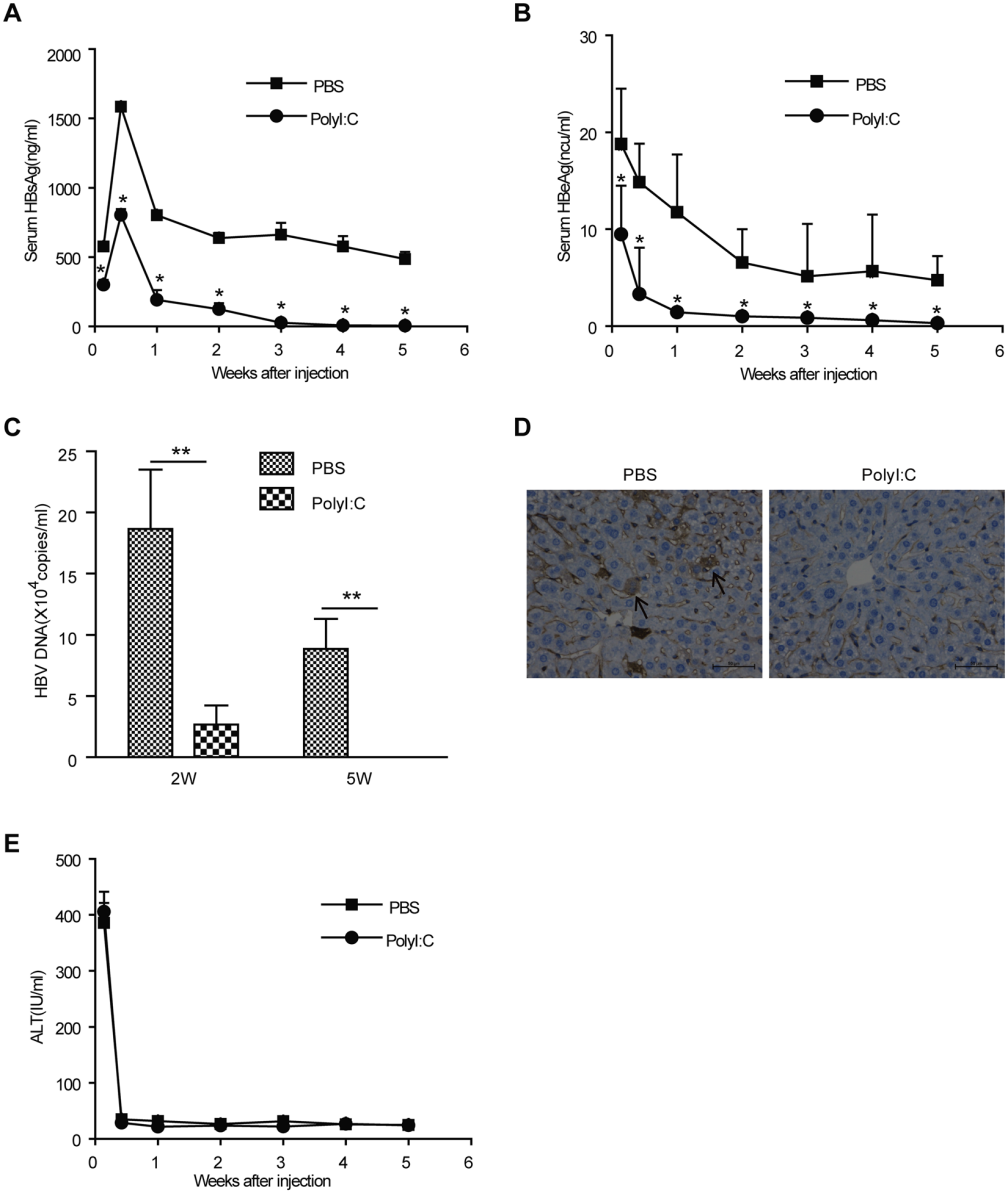
Early PolyI:C treatment prevents HBV persistence in the HBV mouse model. C57BL/6 mice were injected with HBV plasmid and chronically treated with Poly I:C as described in Materials and Methods. Titers of serum HBsAg (**A**), HBeAg (**B**), HBV DNA (**C**), and ALT (**E**) were determined at indicated time points. (**D**) At 5 weeks post injection, Livers were processed for HBcAg expression (original magnification, x100). Data are shown as mean ± SEM; *p < 0.05, **p < 0.01. *n* = 6–8 in each group.

To investigate the effect of NK-cell activation on HBV infection, mice were hydrodynamically injected with pAAV/HBV1.2 and co-treated with PolyI:C. Application of PolyI:C inhibited the initial increase of serum HBsAg and HBeAg levels and eliminated the expression of the two proteins at 5 weeks post injection (Fig. 3A and B). HBV DNA copies in the serum of PolyI:C-treated and mock-treated mice were also assayed. PolyI:C treatment dramatically reduced the level of HBV replication 2 weeks and 5 weeks post pAAV/HBV1.2 injection (Fig. 3C), and HBcAg protein expression in liver tissues of PolyI:C-treated mice could not be detected 5 weeks post injection(Fig. 3D). These data demonstrate that PolyI:C treatment induced clearance of HBV. Additionally, PolyI:C treatment did not increase serum ALT levels (Fig. 3E), indicating that PolyI:C suppressed HBV expression in a noncytolytic process.

### PolyI:C induced clearance of HBV *via* an NK-dependent mechanism

**Figure 4:**
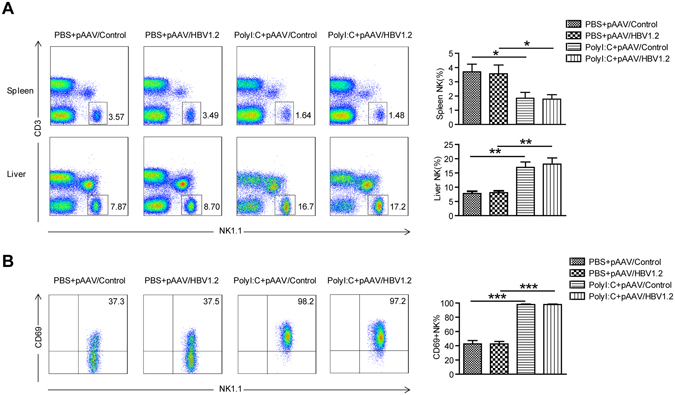
PolyI:C treatment activates NK cells and induces accumulation of NK cells in the liver. Mice were injected with HBV plasmid or control plasmid and chronically treated with Poly I:C as described in Materials and Methods. Lymphocytes were isolated from the liver and spleen and analyzed by flow cytometry. (**A**) The percentages of NK cells (CD3-NK1.1+) were analyzed using anti-NK1.1 and anti-CD3 antibodies. (**B**) The surface expression of CD69 on gated NK cells (CD3-NK1.1+) was also analyzed. Data are shown as mean ± SEM; **p < 0.01, ***p < 0.001. *n* = 4–5 in each group.

**Figure 5:**
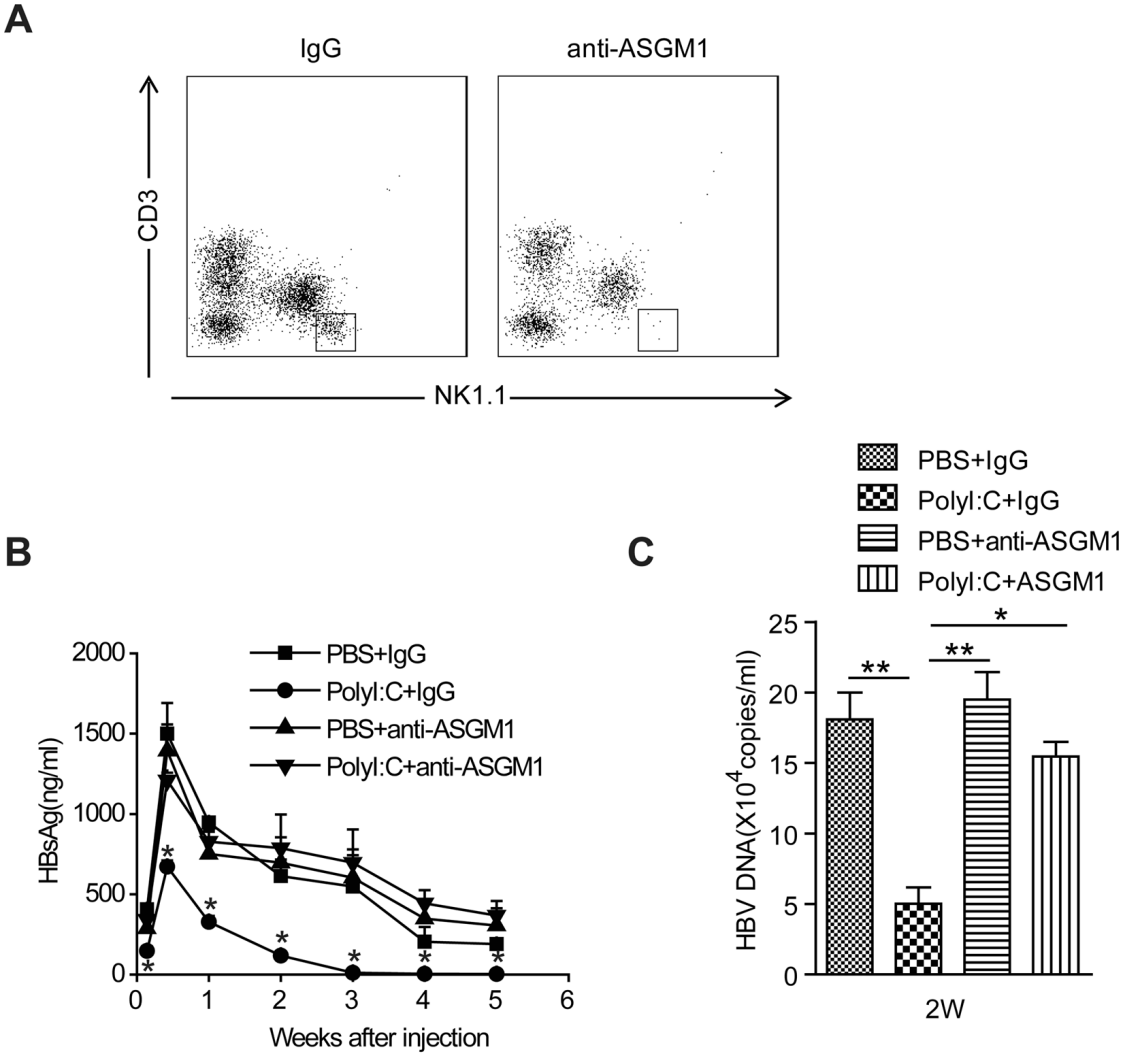
Depletion of NK cells abrogates the anti-HBV activity of PolyI:C. Mice were injected with HBV plasmid and chronically treated with control Ab or anti–ASGM-1 Ab and Poly I:C, as described in Materials and Methods, for 5weeks. (**A**) Depletion of NK cells (NK1.1 + CD3−) was confirmed by flow cytometry. (**B**) Titers of serum HBsAg were determined at the indicated time points post injection. *p < 0.05 vs. PBS + IgG group or PBS + anti–ASGM-1 group or Poly I:C + anti–ASGM-1 group. (**C**) Titers of serum HBV DNA copies were determined at 2 weeks post injection. Data are shown as mean ± SEM; *p < 0.05, **p < 0.01. *n* = 4–6 in each group.

Next, we investigated whether the anti-HBV activity of PolyI:C was due to the effect of PolyI:C on NK cells. We found that NK cells were dramatically increased in the liver but significantly decreased in the spleen following PolyI:C treatment, regardless of whether the treated mice were HBV-bearing or not (Fig. 4A). The expression of activation marker CD69 on hepatic NK cells was also enhanced in PolyI:C-treated mice (Fig. 4B). So, injection with PolyI:C caused recruitment and strong activation of NK cells in the liver. To further elucidate the role of NK cells, anti-ASGM1 Ab was used to deplete NK cells (Fig. 5A). As shown in Fig. 5B, NK cell depletion potently impaired HBV clearance and resulted in persistently maintained serum HBsAg in PolyI:C treated mice. Serum HBV DNA levels also confirmed that NK cell depletion could abrogate the anti-HBV activity of PolyI:C(Fig. 5C). These findings suggest NK cells played a critical role in PolyI:C-induced anti-HBV activity.

### Anti-HBV activity of PolyI:C is dependent on NK cell-derived IFN-γ

**Figure 6:**
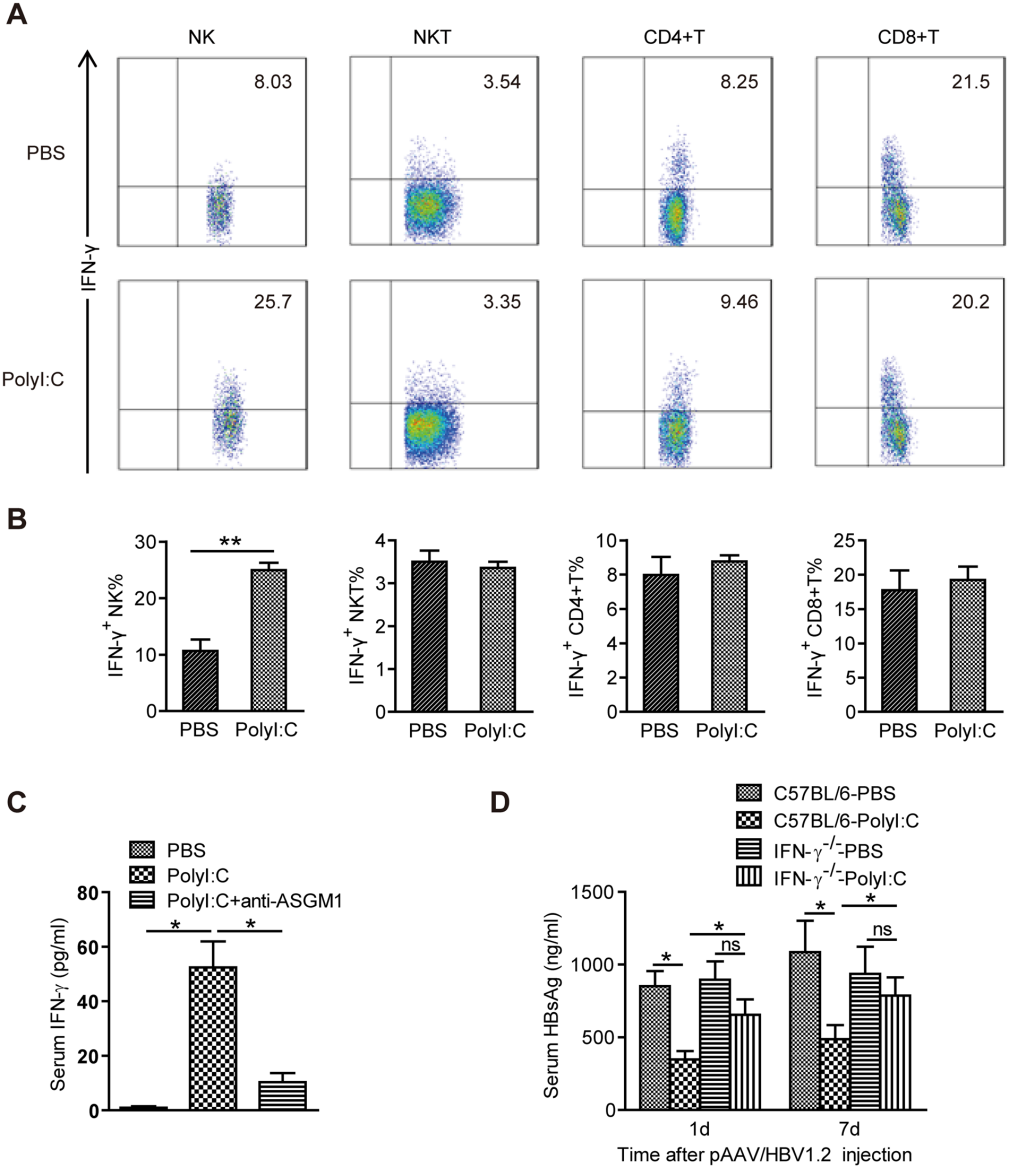
Anti-HBV activity of PolyI:C is dependent on NK cell-derived IFN-γβ. (**A**,**B**) C57BL/6 mice were injected with HBV plasmid and chronically treated with Poly I:C as described in Materials and Methods. Intracellular cytokine staining was performed to examine IFN-γ production from NK cells (CD3-NK1.1+) at 6 days after pAAV/HBV1.2 injection. (**C**) Mice were injected with HBV plasmid and chronically treated with anti–ASGM-1 Ab and Poly I:C as described in Materials and Methods. Serum levels of IFN-γ at 6 days after pAAV/HBV1.2 injection were determined by ELISA. (**D**) IFN-γ^−/−^ mice were hydrodynamically injected with HBV plasmid and chronically treated with Poly I:C. Titers of serum HBsAg were determined at the indicated time points post injection. Data are shown as mean ± SEM; *p < 0.05, **p < 0.01. *n* = 4–6 in each group.

IFN-γ is known to inhibit HBV gene expression noncytopathically^20^. As shown in Fig. 6A and B, PolyI:C treatment increased intracellular expression of IFN-γ by NK cells as well as circulating serum IFN-γ levels. Depletion of NK cells significantly decreased serum IFN-γ levels(Fig. 6C), suggesting that IFN-γ was produced by NK cells. To examine the role of IFN-γ in PolyI:C -induced antiviral activity, we monitored the ability of PolyI:C to inhibit HBV gene expression in IFN-γ^−/−^ mice (mice deficient in IFN-γ). As shown in Fig. 6D, IFN-γ deficiency led to a significant increase in expression of HBsAg in PolyI:C-treated mice, suggesting that IFN-γ was required for the anti-HBV effect of PolyI:C.

### PolyI:C therapy leads to NK cell-dependent HBV suppression in HBV-tolerant mice and HBV-transgenic mice

**Figure 7:**
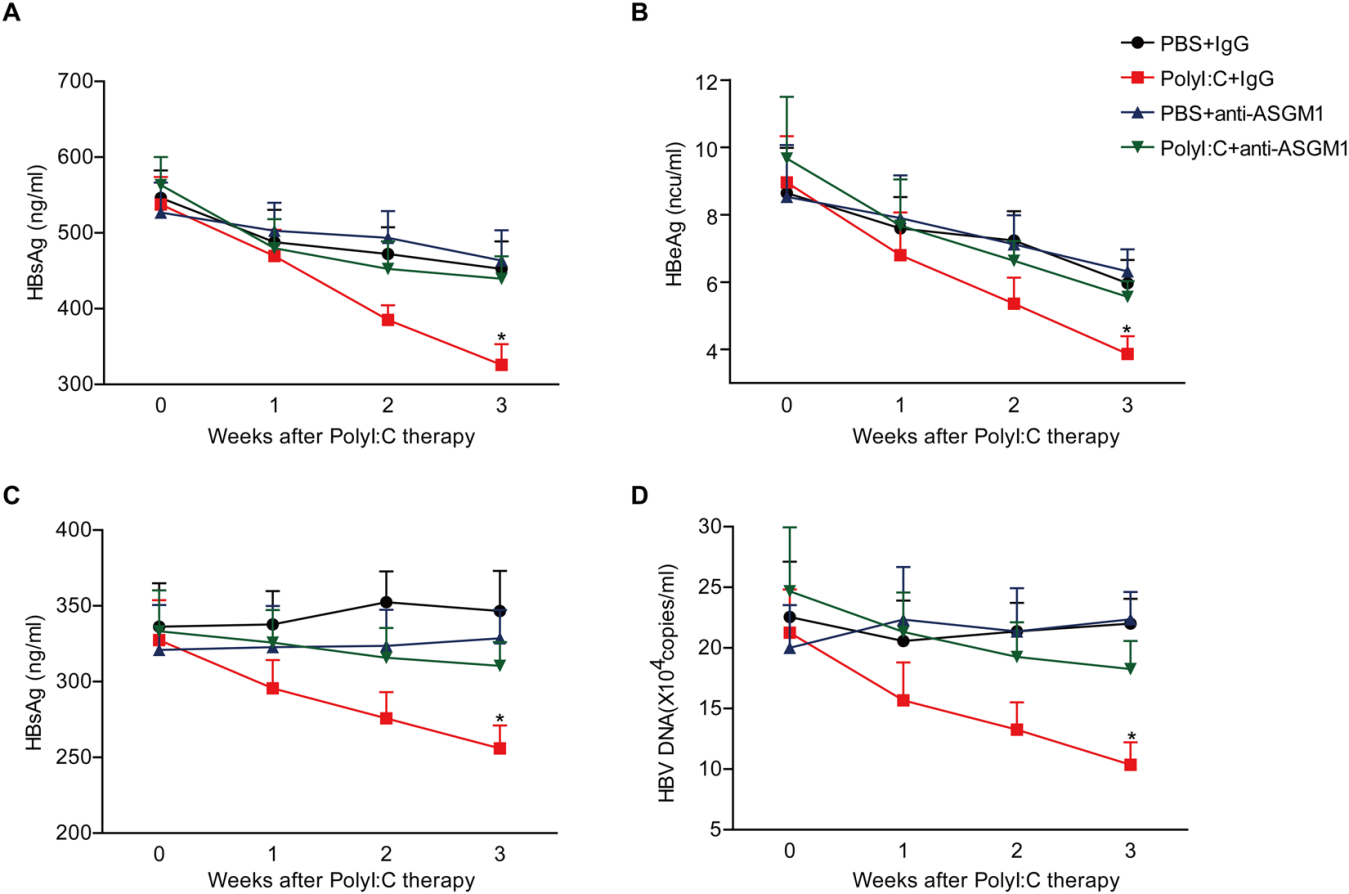
PolyI:C therapy leads to NK cell-dependent anti-HBV activity in HBV-tolerant mice and HBV-transgenic mice. (**A**,**B**) C57BL/6 mice were injected with HBV plasmid, 2 weeks later, HBV-tolerant mice were chronically treated with control Ab or anti–ASGM-1 Ab and Poly I:C, as described in Materials and Methods, for 3 weeks. Titers of serum HBsAg (**A**) and HBeAg (**B**) were determined at the indicated time points post injection. *p < 0.05 vs. PBS + IgG group or PBS + anti–ASGM1group or Poly I:C + anti–ASGM-1group. Data are shown as mean ± SEM; *n* = 5 in each group. (**C**,**D**) HBV-transgenic mice were chronically treated with control Ab or anti–ASGM-1 Ab and Poly I:C, as described in Materials and Methods, for 3 weeks. Titers of serum HBsAg (**C**) and HBV DNA (**D**) were determined at the indicated time points post injection. *p < 0.05 vs. PBS + IgG group or PBS + anti–ASGM1group or Poly I:C + anti–ASGM-1group. Data are shown as mean ± SEM; *n* = 3–5 in each group.

Next, we examined whether activation of NK cells by PolyI:C could also suppress HBV in HBV-tolerant mice and HBV-transgenic mice. C57BL/6 mice received hydrodynamic injection of pAAV/HBV1.2 and then PolyI:C or PBS 2 weeks later. As shown in Fig. 7A and B, serum levels of HBsAg and HBeAg significantly decreased in HBV-tolerant mice after 3 weeks post PolyI:C therapy, but depletion of NK cells by anti-ASGM1 impaired the anti-HBV activity of PolyI:C, suggesting that NK cells were implicated in PolyI:C therapy for chronic HBV infections. Furthermore, PolyI:C therapy could also suppress HBsAg expression and HBV replication in HBV-transgenic mice, and this is dependent on NK cells (Fig. 7C and D). These data indicate that activation of NK cells by PolyI:C might have therapeutic potential for the treatment of chronic HBV-infected patients.

### Kupffer cells (KCs) are required for the anti-HBV activity of NK cells induced by PolyI:C in the mouse model

**Figure 8:**
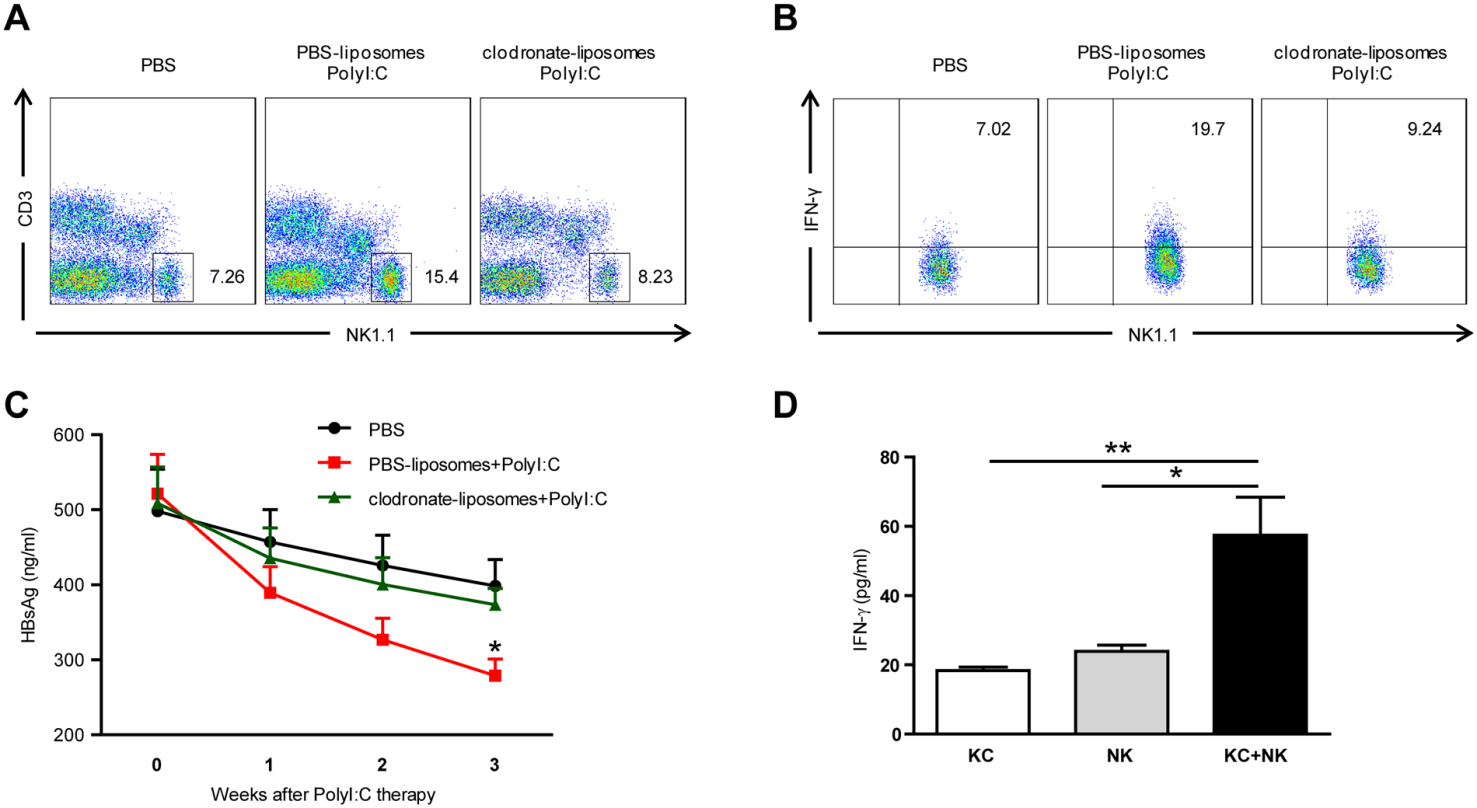
The presence of KCs is necessary for NK cell anti-HBV activity in PolyI:C-treated mice. (**A**,**B**,**C**) C57BL/6 mice were injected with HBV plasmid, 2 weeks later, HBV-tolerant mice were chronically treated with PBS or PBS-liposomes + PolyI:C or clodronate-liposomes + PolyI:C, as described in Materials and Methods, for 3 weeks. (**A**) The percentages of NK cells (CD3-NK1.1+) were analyzed using anti-NK1.1 and anti-CD3 antibodies. (**B**) Intracellular cytokine staining was performed to examine IFN-γ production from NK cells (CD3-NK1.1+). (**C**) Titers of serum HBsAg were determined at the indicated time points post injection. *p < 0.05 vs. PBS or clodronate-liposomes + PolyI:C. Data are shown as mean ± SEM; *n* = 5 in each group. (**D**) Hepatic NK cells were cultured alone or cocultured with KCs at a ratio of 1:1 and stimulated by poly I:C (100 μg/mL). After 48 h, IFN-γ secretion in the supernatant was quantified by ELISA. Data are expressed as mean ± SEM; *p < 0.05. **p < 0.01.

KCs, the resident macrophage population of the liver, are poised to initiate innate immune responses by secretion of cytokines and direct cellular contact when primed by pathogen-derived products^21^. To test whether KCs might be involved in the PolyI:C-induced NK cell response, we depleted KCs by clodronate-liposomes. Successful KC depletion was verified by flow cytometry analysis of reduction in F4/80+ cells as described before (data not shown)^22^. Depletion of KCs significantly inhibited the accumulation of NK cells in the liver (Fig. 8A), and suppressed the secretion of IFN-γ by NK cells (Fig. 8B), suggesting that KC were involved in the NK cell response. Accordingly, PolyI:C-induced HBV suppression was impaired in KC-depleted mice (Fig. 8C), indicating that KCs were indispensable for HBV suppression in PolyI:C-treated mice. Further *in vitro* data also showed that PolyI:C could not directly stimulate NK cells to secrete IFN-γ; however, when co-cultured with PolyI:C-stimulated KCs, NK cells were able to produce IFN-γ (Fig. 8D). These results suggested that NK cell anti-HBV activity depends on the presence of KCs in PolyI:C-treated mice.

## Discussion

In the present study, a mouse model for HBV persistence was generated by hydrodynamically injecting an HBV genome-containing plasmid (pAAV/HBV1.2) into mice. Early application of PolyI:C markedly decreased serum HBV levels and prevented HBV persistence. Also, PolyI:C therapy led to HBV suppression in HBV-tolerant mice and HBV-transgenic mice. We found that PolyI:C -induced anti-HBV activity was dependent on NK cells and IFN-γ, which is an previously undescribed anti-HBV mechanism of PolyI:C.

NK cells are abundant in the normal liver and may play a critical role in immune responses to infections of this organ^5^. In this study, we did not observe any difference in the abundance or activation of the NK cell populations in the livers of HBV-injected versus control mice, supporting the view that HBV is capable of sneaking through the front line of host defenses.

Toll-like receptors (TLRs) sense pathogen-associated molecule patterns(PAMPs) and activate antiviral mechanisms, including intracellular antiviral pathways and the production of antiviral cytokines, resulting in a suppression of HBV replication and spreading^23, 24^. Nucleic acids derived from pathogens, including single- or double-stranded RNAs, represent a major class of PAMPs^25^. The synthetic analogue of double-stranded RNA Poly I:C, which might reflect a natural genetic product from a variety of viruses during replication, can be sensed by TLR3 and melanoma differentiation-associated gene 5 (MDA-5)^26^. We showed here that early Poly I:C treatment could reduce HBV antigen loads and prevent HBV persistence in mice, indicating that vigorous innate immune responses in the early phase of HBV infection might inhibit initial HBV replication and gene expression and eventually promoted viral clearance.

Apart from its direct antiviral activity, Poly I:C also activated immune cells. It has been shown to enhance the cytotoxicity of NK cells and macrophages and to activate T cells^27^. In this paper, we presented that NK cells were dramatically recruited into and activated in the liver following PolyI:C injection. Furthermore, depletion of NK cells abrogated the anti-HBV activity of PolyI:C, suggesting that intrahepatic NK cells might control HBV infection if they become activated.

Notably, our results demonstrated that NK cell–mediated anti-HBV activity induced by Poly I:C was dependent on cooperation with KCs. Both *in vivo* and *in vitro* data showed that KCs were critical for NK cell–derived IFN-γ production. It was shown that human KCs might activate NK cells through IL-12 and IL-18 and direct cell contact in response to TLR ligands stimulation^21^. IL-12 has been reported to be involved in promoting NK cell accumulation in the liver^28^. TNF-α, another cytokine released mainly by activated macrophages, could also recruit NK cells to liver and enhance NK cell IFN-γ production *in vitro* and *in vivo* ^29, 30^. These study may be helpful for further analysis of the mechanisms KCs use to activate NK cells in our model.

As to date, attempts to therapeutically restore HBV–specific T cell responses in chronically HBV-infected patients have not been very successful, probably due to functional exhaustion or physical deletion of virus-specific T cells^31^. In contrast, since NK cells do not recognize viral peptide antigens, they are less likely to be affected by high antigen loads. Even HBV is poorly sensed by the innate immune system, NK cells remain well-poised to respond to HBV infection given the very low basal expression of MHC class I by liver cells^32^ and given the high frequency of NK cells in the liver, where NK cells make up approximately 30% of human intrahepatic lymphocytes. Thus, it may be possible to use pharmacological approaches to activate the intrahepatic NK cell population, thereby rapidly and efficiently enhancing the production of antiviral cytokines in the liver of infected patients. Previous studies have found that activation of NKT cells, another major innate immune cell in liver, could inhibit HBV replication *in vivo* ^33, 34^. These findings, together with our own, support the view that activating innate immune responses may be a therapeutic approach to compensate for the failure of acquired immune system to treat chronic HBV infection.

Previous studies have found that HBV proteins like HBxAg protein^35, 36^, polymerase^37^ can interfere with innate immunity, thereby attenuating the antiviral response of the innate immune system and ultimately contributing to the establishment of persistent infections. The liver receives 80% of its blood supply from the gut through portal vein, as a result, the liver is continuously exposed to gut microbial products which might activate innate immunity during primary HBV infection^38^. Our results suggest that outcomes of HBV infection might be influenced by the initial activation and strength of the immune response during the early phase of HBV infection in human beings.

In conclusion, this study demonstrates that PolyI:C activates NK cells to secrete antiviral cytokine IFN-γ and induces accumulation of NK cells in the liver. Early PolyI:C treatment leads to NK cell-dependent clearance of HBV in the mouse model; activation of NK cells by PolyI:C therapy could also suppress HBV in HBV-tolerant mice and HBV-transgenic mice. Based on these results, it is conceivable that NK cells are implicated in host defense against HBV during natural infection. In addition, the results suggest that pharmacological activation of NK cells might have a good potential for treating chronic hepatitis B.

## Materials and Methods

### Animals

Male C57BL/6 mice (5–7 weeks old) were obtained from and raised at Chongqing Medical University. HBV transgenic mice were obtained from Infectious Disease Center of No. 458 Hospital (Guangzhou, China). IFN-γ^−/−^ mice were purchased from Model Animal Research Center (Nanjing, China). All mice were housed under specific pathogen-free conditions and used according to the regulations of animal care of Chongqing Medical University. The study protocol was approved by the ethical committee of the Second Affiliated Hospital of Chongqing Medical University and the methods were carried out in accordance with the approved guidelines.

### Plasmids injection and PolyI:C treatment

pAAV/HBV1.2 plasmid was kindly provided by Dr. Pei-Jer Chen^18^. pAAV/Control plasmid was used as a control plasmid for pAAV/HBV1.2. The plasmids were isolated by using an endotoxin-free kit (Qiagen, Inc.). Hydrodynamic injection of the pAAV/HBV1.2 or pAAV/Control plasmid into mice was performed as described^39, 40^.

Early PolyI:C treatment. Mice were injected intravenously with 5 μg/g bodyweight poly I:C (Sigma, St. Louis, MO) 24 hours before 6 μg pAAV/HBV1.2 injection and then treated with poly I:C every 48 hours for 3 weeks. Control mice received pAAV/HBV1.2 plasmid plus saline injection.

PolyI:C therapy. C57BL/6 mice were hydrodynamically injected with 6 μg pAAV/HBV1.2 plasmid, 2 weeks later, HBV-tolerant mice were injected intravenously with 5 μg/g bodyweight poly I:C every 48 hours for 3 weeks. Control mice received pAAV/HBV1.2 plasmid plus saline injection.

### Detection of HBV antigen and liver transaminase activities

Serum levels of HBsAg, HbeAg were determined with commercially available radioimmunoassay (RIA) kits (Beijing North Institute of Biological Technology, Beijing, China). Serum enzyme activities of alanine aminotransferase (ALT) were measured using commercially available kit (Rong Sheng, Shanghai, China).

### HBV DNA detection

Serum HBV DNA copies were assessed by quantitative PCR using a commercial kit for HBV DNA (AMPLLY, Xiamen, China).

### Immunohistochemistry

HbcAg+ hepatocytes in liver tissue were examined by immunohistochemistry as previously described^40^.

### Cell preparations and fluorometric analysis

Splenocytes and liver mononuclear cells (MNCs) were isolated essentially as described previously^41^. Abs against the following proteins were used: anti-NK1.1 (PK136), anti-CD3 (145-2C11), anti-CD69 (H1.2F3), anti-IFN-γ (XMG1.2), anti-F4/80 (BM8) (all of above from eBioscience). Cells in a single-cell suspension were stained with fluorescence-labeled mAbs for surface antigens according to a standard protocol. To detect IFN-γ expression, hepatic MNCs were stimulated with PMA (30 ng/ml; Sigma-Aldrich) and ionomycin (1 µg/ml; Sigma-Aldrich) and monensin (5 µg/ml; Sigma-Aldrich). After 4 h of culture at 37 °C and 5% CO_2_, the cells were harvested and labeled by anti-NK1.1, anti-CD3. Then the cells were fixed, permeabilized, and stained with anti-IFN-γ. Data were collected on a BD FACSCanto^TM^ II cytometer and analyzed using FlowJo analysis software 7.6.1(Tristar).

### Cell depletion

To deplete NK cells, mice were intravenously injected with 30 μg of anti-ASGM1 (Wako, Richmond, VA) or control rabbit IgG per mouse 24 hours before challenge. To chronically deplete NK cells, mice were treated with anti-ASGM1 every 3 days. For KC depletion, mice were injected intravenously with 100 μl of clodronate-liposomes or PBS-liposomes (provided by Dr. N. van Rooijen, Vrije Universiteit, Amsterdam, The Netherlands) 48 hours before challenge. To chronically deplete KCs, mice were treated with clodronate-liposomes every 1week. The elimination of NK cells and KCs was confirmed by flow cytometry.

### ELISA

Serum levels of IFN-γ were assessed using a ELISA kit (Dakewei, Beijing, China), according to the manufacturer’s instructions.

#### Isolation of NK Cells and KCs

Liver NK cells were separated by negative magnetic cell sorting using the NK Cell isolation kit II according to the manufacturer’s protocol (Miltenyi Biotec, Auburn, CA, USA). Approximately 90% of the magnetic cell sorting-purified cells were NK1.1 + CD3−.

Single cell suspensions of hepatic non-parenchymal cells (NPCs) were prepared using two-step collagenase perfusion method as described before^42^. KCs were enriched by positive magnetic cell sorting using phycoerythrin (PE)-conjugated anti-F4/80 mAb and anti-PE-MicroBeads according to the manufacturer’s protocol (Miltenyi Biotec, Auburn, CA, USA). The separated cells were ≥85% pure.

#### Culture of hepatic NK cells and KCs

Purified NK cells were cultured alone or co-cultured with KCs at 1:1 ratio in RPMI-1640 complete medium for 48 hours. PolyI:C was used at a final concentration of 100 μg/ml. IFN-γ secretion in the supernatant was quantified by ELISA.

### Statistical analysis

All data are expressed as the mean and standard error of the mean (SEM). Results were analyzed by using Student’s *t* test or ANOVA where appropriate. *P* < 0.05 was considered statistically significant for all tests.
